# Diagnosis of vertebral column pathologies using concatenated resampling with machine learning algorithms

**DOI:** 10.7717/peerj-cs.547

**Published:** 2021-07-22

**Authors:** Aijaz Ahmad Reshi, Imran Ashraf, Furqan Rustam, Hina Fatima Shahzad, Arif Mehmood, Gyu Sang Choi

**Affiliations:** 1 College of Computer Science and Engineering, Department of Computer Science, Taibah University, Al Madinah Al Munawarah, Saudi Arabia; 2 Information and Communication Engineering, Yeungnam University, Gyeongbuk, Gyeongsan-si, South Korea; 3 Department of Computer Science, Khwaja Fareed University of Engineering and Information Technology, Rahim Yar Khan, Pakistan; 4 Department of Computer Science & Information Technology, The Islamia University of Bahawalpur, Bahawalpur, Pakistan

**Keywords:** Data classification, Biomedical parameters, Data resampling, Machine learning, Pathology diagnosis

## Abstract

Medical diagnosis through the classification of biomedical attributes is one of the exponentially growing fields in bioinformatics. Although a large number of approaches have been presented in the past, wide use and superior performance of the machine learning (ML) methods in medical diagnosis necessitates significant consideration for automatic diagnostic methods. This study proposes a novel approach called concatenated resampling (CR) to increase the efficacy of traditional ML algorithms. The performance is analyzed leveraging four ML approaches like tree-based ensemble approaches, and linear machine learning approach for automatic diagnosis of inter-vertebral pathologies with increased. Besides, undersampling, over-sampling, and proposed CR techniques have been applied to unbalanced training dataset to analyze the impact of these techniques on the accuracy of each of the classification model. Extensive experiments have been conducted to make comparisons among different classification models using several metrics including accuracy, precision, recall, and *F*_1_ score. Comparative analysis has been performed on the experimental results to identify the best performing classifier along with the application of the re-sampling technique. The results show that the extra tree classifier achieves an accuracy of 0.99 in association with the proposed CR technique.

## Introduction

The vertebral column is an important part of the human axial skeleton system. The vertebral column is composed of about 33 bones known as vertebrae. The vertebrae in the column are separated by intervertebral discs. The vertebral column encases the spinal cord within the spinal canal formed by these bones (vertebrae) and intervertebral discs. The encasing of the spinal cord protects the human and supports by carrying the above pelvis weight. It also plays the role of the central axle and facilitates postures and movement (Marks, 2019; DeSai, Vamsi & Agarwal, 2018). The vertebral column can be affected by pathological changes due to aging or trauma because of fatigue or mechanical overload. The consequences of the pathology can lead to back pain of different intensities and pain or numbness at other locations in the human body (Raciborski, Gasik & Kłak, 2016). The common pathologies of the vertebral column include osteoarthritis, osteoporosis, seronegative spondyloarthropathies, spinal cord injury, spinal disc herniation, spondylolisthesis, and spinal stenosis (DeSai, Vamsi & Agarwal, 2018; Fardon & Milette, 2001). Such problems can cause pain in the lower back and neck which worsens with the age and may require surgery and other medication. Timely and accurate diagnosis of pathologies is an important task in bioinformatics.

Machine learning can play a key role in the field of bioinformatics. In recent years machine learning has aided the practitioners in medical diagnosis, thus facilitating significant improvements in clinical decision support systems. The machine learning models are applied both in disease diagnosis and in prognosis. The models predict a specific label based on the input data. These models can be applied to a variety of data sets in clinical applications, such as medical imaging, bio-potential measurements, physiological parameters, etc (Chen, Liu & Peng, 2019). Due to the increased relevance of machine learning techniques in health care, extensive research and development efforts are required from both academia and industry. The machine learning approaches still face application restriction in the current clinical applications concerning lack of optimality in most of the present systems (Wiens et al., 2019). To overcome this limitation, the gap among the researchers, medical practitioners, and other stakeholders need to be bridged. Machine learning techniques are capable of converting past clinical measurements into important conclusions. These conclusions can then be used to take the essential measures to identify the disease threat.

The current study aims at applying ML models to diagnose the vertebral column pathologies. To this end, four ML models including Adaboost (AB), random forest (RF), extra trees classifier (ETC), and logistic regression (LR) are applied. The goal is to diagnose the two most common vertebral column pathologies spinal disc herniation, and spondylolisthesis. Resampling approaches are leveraged to improve the limited accuracy of traditional ML approaches. The performance of these models with different resampling techniques has been validated and the most appropriate model has been selected given a specific scenario in terms of the nature of data and diagnostic requirements. The key highlights of this study can be summarized as:

- Application of machine learning algorithms to diagnose orthopedic diseases, such as spinal disc herniation, and spondylolisthesis. Four ML algorithms are implemented including AB, RF, ETC, and LR for the diagnosis.
- The impact of resampling approaches is analyzed to enhance the accuracy of the ML algorithms. Two resampling approaches like under-sampling and over-sampling are implemented with the selected ML algorithms and their efficacy is investigated on an imbalanced dataset.
- A novel resampling approach called concatenated resampling is proposed for improving the accuracy of ML algorithms when trained with an imbalanced dataset. Performance analysis is carried out to show the supremacy of the proposed CR approach.
- Performance of the proposed approach is compared with several state-of-the-art approaches in medical diagnosis. Results indicate that the proposed approach outperforms other approaches.

The rest of the paper is organized in the following fashion. The ‘Related Work’ section reviews several research works that are related to the current study. A brief description of vertebral column pathologies is given in the ‘Vertebral Column Pathologies’ section. ‘Radiological Diagnostic Parameters’ describes the pathological parameters that are used in the diagnosis of vertebral pathologies. ‘Materials and Methods’ presents a description of the ML approaches used in the current study, as well as, the resampling approaches and the dataset used for experiments. Additionally, the adopted methodology is also discussed in the same section. ‘Results and Discussion’ contains results and discussion and finally, the conclusion and future work are given in the ‘Conclusion’ section.

## Related work

The significant increase in the application of machine learning-based classification in health care attracted increased interest. Several research works can be found in the literature that adopts ML approaches for serving higher accuracy for prediction and diagnosis (Wiens et al., 2019). For example, the authors used a feature-based approach with RF for diagnosing ten different diseases in Alam, Rahman & Rahman (2019). Promising results indicate that ML approaches are suitable for disease diagnosis tasks.

### Disease prediction using ML classifiers

Rajpurkar et al. (2017) developed an algorithm to detect pneumonia patients using the chest X-rays. The proposed algorithm called CheXNet is a neural network and consists of 121 layers including input/output and convolutional layers. The chest x-ray dataset which is used for training and testing the neural network contains 100,000 x-ray-images with fourteen different diseases. The study shows promising results in the diagnosis of the different thoracic pathologies including pneumonia. Similarly, the performance of various machine learning classification algorithms has been analyzed in Wroge et al. (2018) for their capability to predict Parkinson’s Disease. The work is based on the model construction and validation using a voice dataset collected from both normal persons as well as persons with Parkinson’s disease. Thus the study presented the effectiveness of the applicability of supervised classification models in the diagnosis of Parkinson’s disease with an accuracy of 85%. Despite the dataset used in the study is high dimensional and contained noise, the prediction accuracy is good.

The efficacy of various statistical and machine learning models is investigated in Huang et al. (2017) for diagnosing breast cancer. In particular, support vector machines (SVM) have been explored and their high performance is reported against other machine learning classifiers. Similarly, Samb et al. (2012) performed experiments on various medical datasets and proposed a modified RFE-SVM model. The ionosphere, the SpamBase, and the SPECTF Heart datasets from the UCI repository have been used in the study. To improve the performance of the final classifier, the RFE-SVM algorithm has been combined with local operators which showed significant classification enhancement.

An ensemble machine learning model is proposed in Mandal (2015) to increase the accuracy of traditional machine learning classifiers. The ensemble aims at enhancing the classification accuracy for spine diagnosis. The Bayesian network is used along with the Tabu search algorithm as a classifier. Haar wavelets are used as the projection filter for attribute ranking and relevant feature selection. Results have been compared with SINPATCO platform methods (MLP, GRNN, SVN). The comparative analysis reveals that the ensemble model beats the traditional machine learning models in classification.

Besides the disease prediction from various features extracted from numeric and image data, several researchers focus on using the ML classifiers for biological studies. For example, the authors identify the antioxidant candidates which are influential to maintain health (Ho Thanh Lam et al., 2020). A machine learning-based model is proposed to predict the antioxidant candidates using a benchmark set of sequencing data. Results using 10-fold cross-validation show that RF can identify antioxidant proteins with the highest accuracy of 84.6%. Similarly, Le et al. (2020a) devised an eXtreme Gradient Boosting (XGBoost) model to identify Methylguanine-DNA methyltransferase (MGMT) status in patients with IDH1 glioblastomas. The study uses 53 patients with proven GBM to obtain biomedical features using multimodality MRI for improving the model’s performance, and F-score analysis is performed to identify important features. A total of nine radionics features are identified which fall under the curve of 0.896. Using these identified features, the XGBoost achieves the highest predictive performance and stability with an accuracy of 0.887 and an AUC of 0.896.

### Use of ML classifiers for pathology disease

Besides the above-mentioned studies, several researchers focus on methods that are related to pathology diagnosis. For example, Seera & Lim (2014) developed an intelligent system for classification for the diagnosis of diabetes, liver disorders, and breast cancer. Dennis & Muthukrishnan (2014) proposed an efficient prediction system based on the Adaptive Fuzzy System. It is based on the combination of a genetic algorithm and the fuzzy set. The achieved accuracy on several datasets is approximately 77.0% compared to 79.39% of the existing systems for disease classification. Similarly, a strategy based intelligent medical decision system in proposed in Gorunescu & Belciug (2014). The proposed system is composed of several classifiers including Naive Bayes, self-organizing maps, k-nearest neighbor, multilayer perceptron, SVM, genetic algorithm, probabilistic neural network, radial basis function, and neural networks. The system performs classification for breast cancer and liver fibrosis.

K-nearest neighbor and genetic algorithm have been used in Jabbar, Deekshatulu & Chandra (2013) to develop a hybrid system for the classification of heart disease. In this study, three ensemble approaches based on a tree with linear non-ensemble machine learning models are used for the automatic diagnosis of inter-vertebral pathologies. The performance of the model is evaluated using unbalanced datasets. The experiments have been conducted to make comparisons among different classification models. Results indicate that the proposed approach outperforms other machine learning algorithms regarding the accuracy, precision, recall, and *F*_1_ score.

### Predicting vertebral column pathology using ML classifiers

The assistance of machine learning algorithms and models to diagnose the vertebral column pathologies is gaining wide attraction. da Rocha Neto et al. (2011) used biomechanical features dataset containing six features and three types of target data such as disk hernia, spondylolisthesis, and healthy for classification. It aggregated the disc hernia and spondylolisthesis pathologies classes into one pathology class and the health class remained unchanged. The research used an SVM classifier with linear kernel and KMOD (kernel with moderate decreasing). The model is trained using 80% and 40% training data and SVM achieved the highest results with KMOD (Ayat et al., 2001). SVM achieved an accuracy score of 0.859 using KMOD kernel accuracy when trained on 80% training data. Similarly, the authors developed an LMT (logistic model tree) for automatic prediction of vertebral column pathologies in Karabulut & Ibrikci (2014). The proposed model is the combination of the logistic regression and decision tree. The proposed approach is comprised of preprocessing and classification where preprocessing is the oversampling of data using the SMOTE technique. Later, the classification model LMT is trained using the preprocessed data to achieve higher accuracy of 89.73%.

Along the same direction, Akben (2016) used machine learning algorithms such as KNN, SVM, DT (decision trees), NB (Naïve Bayes), and MLP for vertebral diagnosis. The same six biomechanical features are used and the highest achieved accuracy is 83.22% using the MLP algorithm. Unal & Kocer (2013) also used the MLP and SVM to diagnose the vertebral column pathology and achieved 85.48% accuracy with the MLP model. Alafeef et al. (2019) used the X-ray images to collect five biomechanical features and diagnose the vertebral column pathologies. These features are extracted from the x-ray images of 422 subjects. Subjects are divided into three classes including disc hernia, spondylolisthesis, and normal. To enhance the effectiveness of the classifiers, the data is preprocessed to weight every vertebral feature using a set of weights computed based on Shannon entropy and the fuzzy C-means clustering algorithm. These weighted features are used to train the neural networks model.

The authors proposed using sequential feature selection to select the best features in Prasetio & Riana (2015). Later, hyperparameter tuning is carried out to select the best hyperparameter setting for machine learning models. Among LR, RF, KNN, and XGBoost, KNN achieved the highest accuracy of 93.54%. The ensemble approach tends to increase the classification accuracy, so, an ensemble approach is proposed in Jiménez & Quintero-Ospina (2019). The research uses radial basis, functions neural networks (RBF), SVM, and PNN (probabilistic neural networks). The output of the models is combined using the weighted criteria and the achieved accuracy is 90.2%.

Despite the higher classification accuracy reported in the above-mentioned research works, they are limited by several factors. Predominantly, the dataset contains three target classes where the ratio of data for target classes is not equal. So, the imbalanced dataset affects the accuracy of the classifiers. Similarly, the number of features used in the dataset is small, i.e., only six features. The size of the dataset is also small, comprising only 310 records in total which can affect the classification accuracy. Furthermore, a large number of previous studies use linear and neural networks-based models for vertebral column problem. Such models perform better on binary and large datasets and their performance is compromised when used for multi-class classification on small datasets. To overcome such limitations, the current study considers linear, as well as, tree-based ensemble models on the re-sampled dataset to enhance the classification accuracy. For a summary of the discussed research works, Table 1 is provided.

**Table 1:** Summary of the discussed research works.

Reference	Method	Aim	Features
da Rocha Neto et al. (2011)	SVM classifier, KMOD	Vertebral column pathologies prediction	Biomedical features
Karabulut & Ibrikci (2014)	LMT	Vertebral column pathologies prediction	Biomedical features
Akben (2016)	KNN, SVM, DT, NB and MLP	Vertebral column pathologies prediction	Biomedical features
Alafeef et al. (2019)	Shanon entropy 156 and the fuzzy C-means clustering algorithm and NN	Vertebral column pathologies prediction	X-ray images Biomedical features
Jabbar, Deekshatulu & Chandra (2013)	KNN and genetic algorithm	Vertebral column pathologies prediction	Biomedical features
Mandal (2015)	Ensemble machine learning model	Spine diagnosis	

## Vertebral column pathologies

The vertebral column is composed of a stack of small bones known as vertebrae. These bones are separated by intervertebral discs. The stack goes vertically through the center of our back, from the pelvis to the neck, making a protective tube for our spinal cord (Magee et al., 2015). There are various identified vertebral column diseases, few of them are more common than others. These common vertebral column pathologies include disc herniation and spondylolisthesis.

### Disc herniation

**Figure 1:**
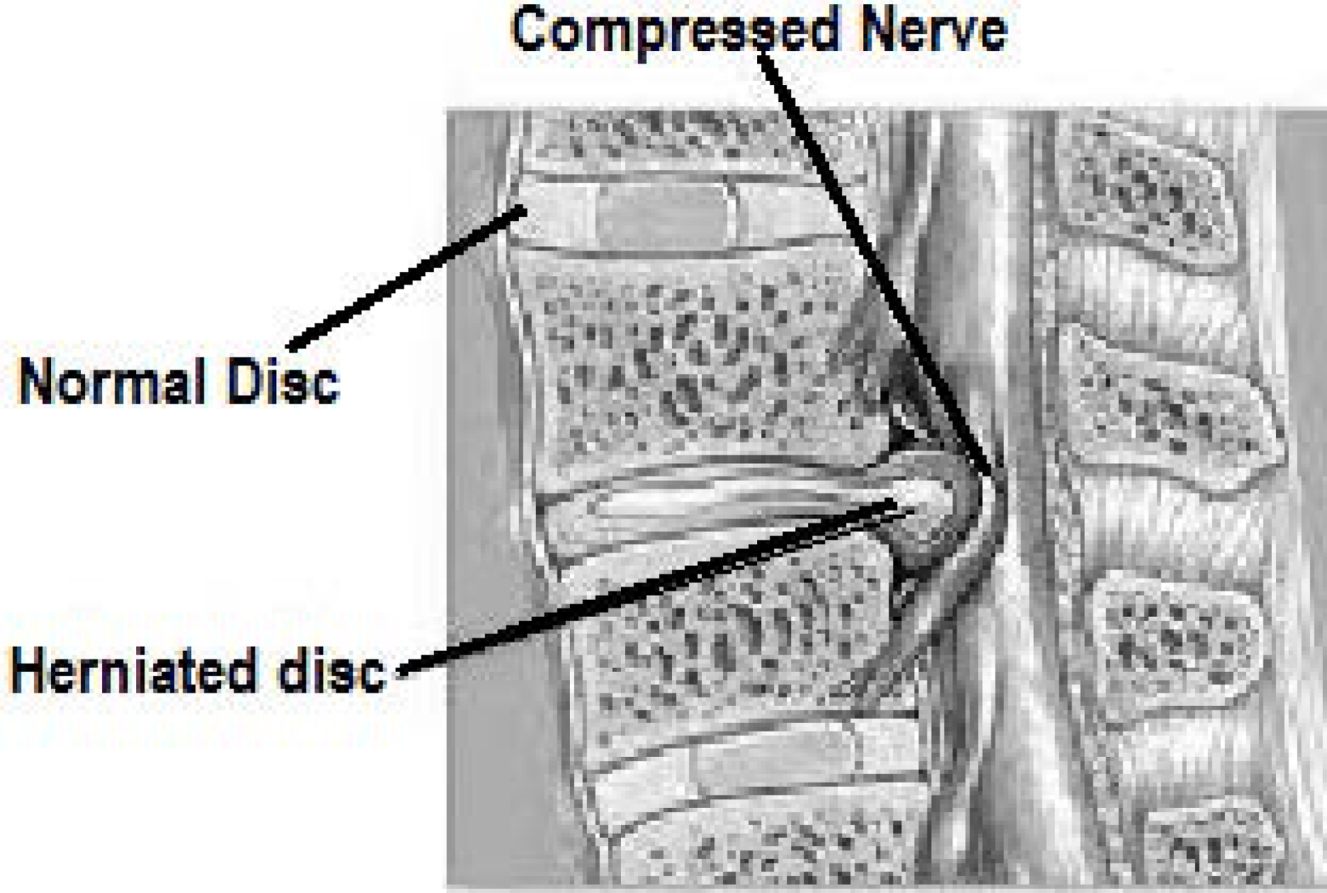
Image showing the disc herniation problem. Due to herniated disc the nerve is compressed.

Intervertebral discs are a fibro-cartilage cushion between two vertebrae in the vertebral column. These discs are made up of the annulus fibrosus, a dense collagenous ring, and nucleus pulposus, a jelly-like material forming the inner core of the vertebrae (Baldit, 2018). Disk herniation is the condition when this jelly-like material (nucleus pulposus) protrudes through the outer ring (annulus fibrosus) of the disc towards spinal nerves as shown in Fig. 1. This process can cause the symptoms of low to severe back pain as well as numbness, or pain in other body parts like legs. These symptoms are caused by the pressure put on the spinal nerves by the protruded nucleus pulposus (Vialle et al., 2010).

### Spondylolisthesis

**Figure 2:**
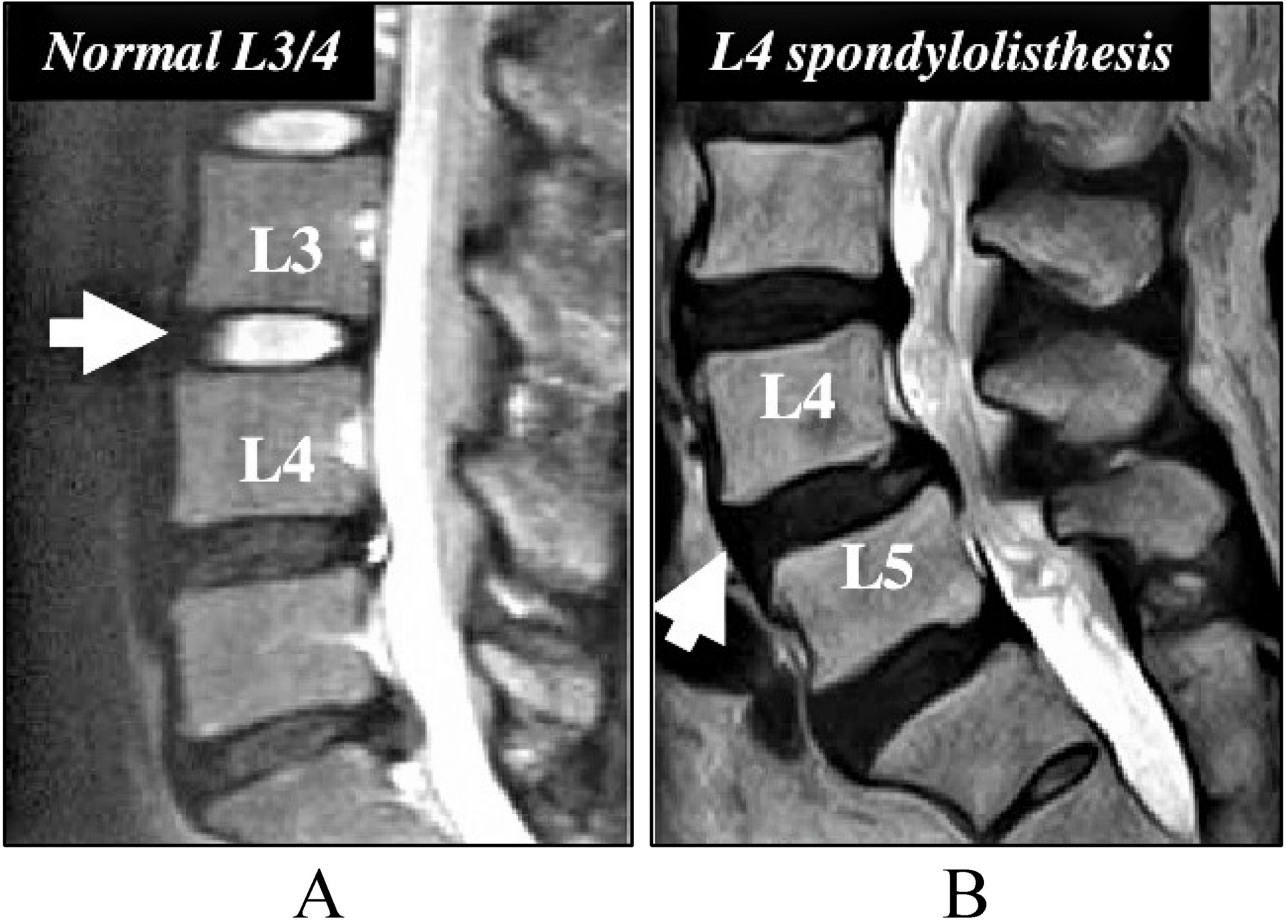
Image for the Spondylolisthesis problem. (A) The normal setting of vertebral body. (B) The spondylolisthesis problem that causes lower back pain.

Spondylolisthesis condition occurs due to the slippage of one vertebral body compared to the other adjacent vertebral body as presented in Fig. 2. This displacement causes symptoms of back pain and can also cause pain in one or both the legs. The grading of illness is based on the degree of slippage of one vertebral body concerning the adjacent vertebral body (Tenny & Gillis, 2019).

## Radiological diagnostic parameters

**Figure 3:**
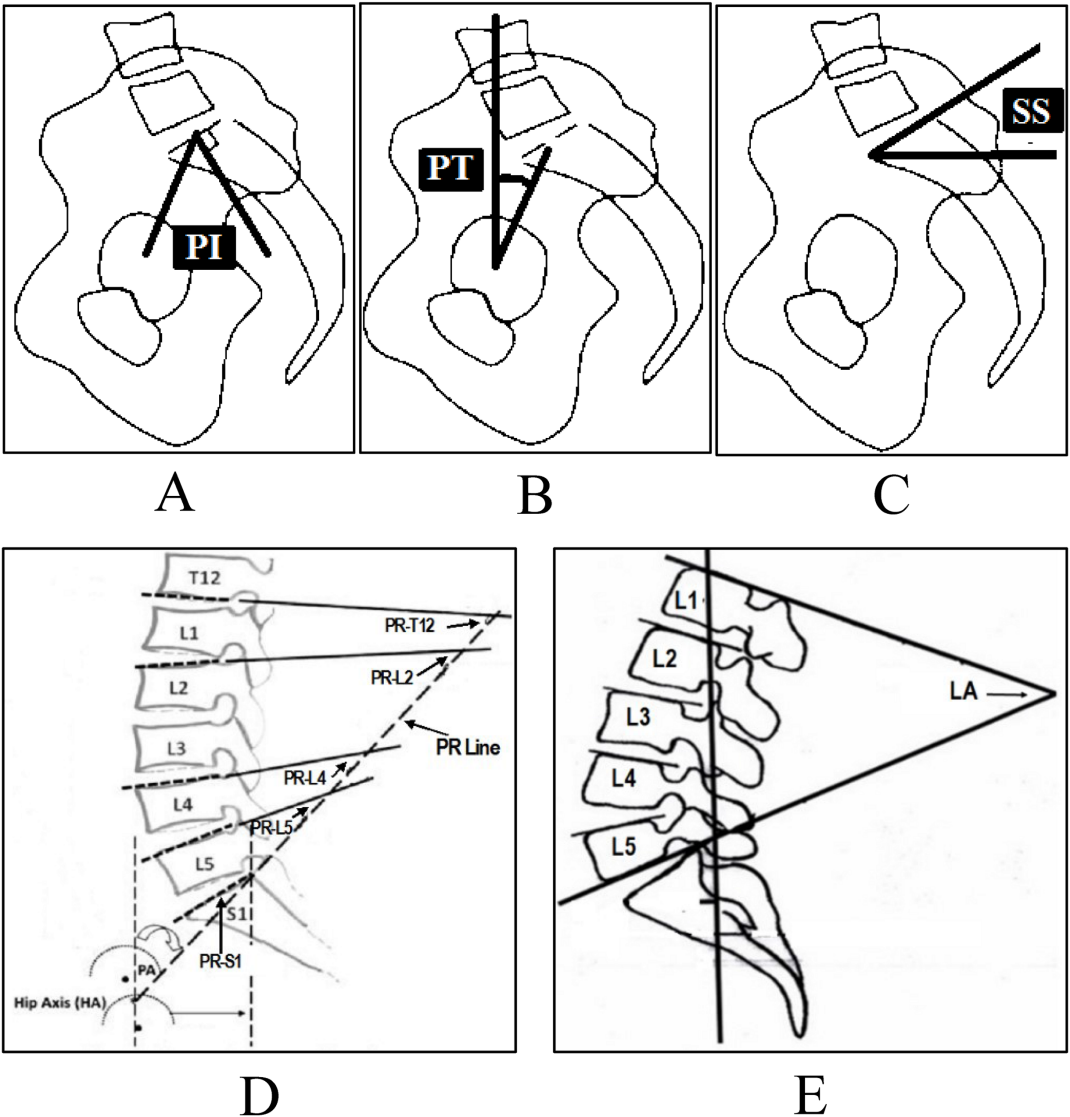
Spinopelvic parameters to diagnose disc herniation and spondylolisthesis, (A) Pelvic Incidence (PI), (B) Pelvic Tilt (PT), (C) Sacral Slope (SS), (D) Pelvic Radius (PR), (E) Lumbar Lordosis (LA).

Several radiological parameters are considered important to diagnose disc problems. For example, pelvic incidence, pelvic tilt and sacral slope, etc. For disc herniation and spondylolisthesis the main radiological parameters used are pelvic incidence (PI) (Fig. 3A), pelvic tilt (PT) (Fig. 3B), sacral slope (SS) (Fig. 3C), lumbar lordosis (LL) (Fig. 3D), pelvic radius (PR) (Fig. 3E) and grade of spondylolisthesis (Léo et al., 2015).

An account of the normal angle-measurement range determined by various authors in several studies (Duarte et al., 2013; Asai et al., 2017) is given in Table 2.

**Table 2:** Normal values for spinopelvic parameters found in the literature.

Parameter	Normal range (in Degrees)
Pelvic Incidence (PI)	40–65
Pelvic Tilt (PT)	10–25
Sacral Slope (SS)	30–50
Lumbar Lordosis (LL)	40–80

## Materials and Methods

### Dataset and tools

The classification efficacy of the proposed approach is investigated using the vertebral column pathology dataset available on the UCI machine learning repository. The dataset is designed by Dr. Henrique da Mota in the Group of Applied Research in Orthopaedics (GARO) of the Centre MAcdico-Chirurgical de RAcadaptation des Massues, Lyon, France (UCI Machine Learning Repository, 2011). Each record in the dataset contains six shapes and orientations of the pelvis and lumbar spine based diagnostic attributes including pelvic incidence, pelvic tilt, sacral slope, lumbar lordosis angle, pelvic radius, and grade of spondylolisthesis. The dataset contains a total of 310 records of individual persons as shown in Table 2, from which 150 are spondylolisthesis patients records and 60 are disc herniation patients. The dataset also contains 100 records of healthy persons without any of the given pathologies. As shown in Table 3, the dataset is not balanced, i.e., the ratio of the number of different class instances is not equal.

**Table 3:** Details of dataset records for each target class.

Records type	Count
Spondylolisthesis patients	150
Disc herniation patients	60
Normal patients	100
Total records	310

### Data re-sampling methods

It is already stated that the dataset that is used in the current study is not balanced concerning records for each class. It contains 310 records, out of which 150 correspond to spondylolisthesis patients (majority class) while only 60 and 150 records correspond to disc herniation patients (minority class) and healthy persons, respectively. The class imbalance in the dataset can result in a significant bias towards the classification of the majority class. It can affect the classification performance adversely while increasing false negatives in the result. To overcome this issue, two types of data re-sampling techniques have been used, i.e., under-sampling, and over-sampling. Further, the cluster centroid technique is considered from the under-sampling category while two techniques synthetic minority and adaptive synthetic are considered from the over-sampling category. Such techniques have been taken regarding their superior performance.

#### Cluster centroids

The cluster centroids (CC) technique has been used for under-sampling in this study. In this method, the majority class is under-sampled by replacing a cluster of majority samples. K-means algorithms are used to find the majority class clusters and then centroids of the *n* clusters are kept as new majority samples (Dataman in AI, 2018). To improve the accuracy of the classification for minority class, this cluster-based method has been used. The impact of undersampling on the accuracy, using CC, has been already investigated in Yen & Lee (2009). The results of this study proved that the cluster-based under-sampling technique helps to achieve higher accuracy than that of other techniques.

#### Synthetic minority oversampling technique

The state-of-art synthetic minority oversampling technique (SMOTE) has been used for over-sampling in this study. The synthetic data instances have been produced by this method using K-nearest neighbors. The over-sampling of the minority class has been done by creating synthetic instances rather than simple replacement. In this way, SMOTE improves the accuracy of classifiers for the minority class. The SMOTE has been already tested on a variety of training datasets with varying imbalances and reported better performance than using other over-sampling techniques (Chawla et al., 2002).

#### Adaptive synthetic

In this study, the adaptive synthetic (ADASYN) technique has been used for over-sampling to analyze its performance against SMOTE. Since ADASYN follows an improved working mechanism of SMOTE, it is expected to show superior performance than that of SMOTE. ADASYN uses a weighted distribution for different minority class examples according to their level of difficulty in learning. After generating a sample, it adds random value, which makes it more realistic and shows effectiveness in classification problems (He et al., 2008). Thus it improves the classification results concerning data distribution by reducing the bias and adaptively shifting the classification decision boundary toward the difficult examples.

#### Concatenated re-sampling

**Figure 4:**
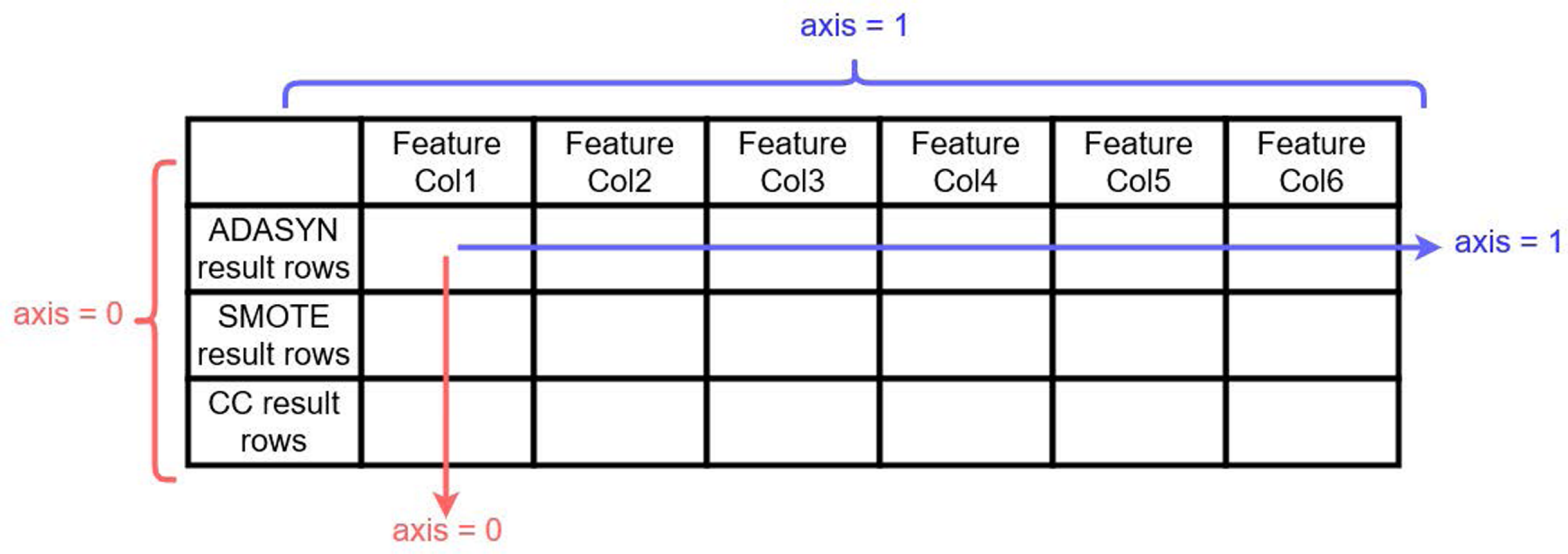
Visualization for ADASYN, SMOTE and CC results concatenation along 0 axis 257.

Together with the above-described re-sampling approaches, experiments have been conducted for the usability of another approach named concatenated re-sampling (CR). In this approach, the results of ADASYN, SMOTE, and CC have been concatenated to improve the results of learning models. The technique results have been concatenated along axis ‘0’ as shown in Fig. 4. CR can thus be defined as:

(1) $$C{R_{(m \times n)}} = [ADASY{N_{(i \times j)}}, SMOT{E_{(u \times v)}}, C{C_{(p \times q)}}]$$

where *ADASYN*_(*i*__×__*j*)_ represents the results of the ADASYN technique, *i* and *j* represents the number of attributes/features and records, respectively. Similarly, *SMOTE*_(*u*__×__*v*)_ and *CC*_(*p*__×__*q*)_ are the results of SMOTE and CC techniques, where *u*, *p* and *v*, *q* representing the number of attributes and number of records, respectively. While *CR*_(*m*__×__*n*)_ is the concatenation results of these three re-sampling techniques where *m* = *i* = *u* = *p* is the number of attributes and *n* = *j* + *v* + *q* is the number of records.

### Machine learning models

The current study considers four machine learning algorithms including AB, RF, ETC, and LR as the classification algorithms for disc pathologies. Their hyperparameters are tuned to enhance their classification accuracy. The list of parameters used in the experiments and their associated values are given in Table 3.

#### Adaboost classifier

AdaBoost an acronym for Adaptive boosting is an ensemble learning meta-model that creates a strong classifier from many weak learners using boosting. By default, the weak learner in the AB classifier is a decision tree with a single split known as a decision stump. The algorithm assigns fewer weights on the already well-handled instances and assigns more weight on the instances which are difficult to classify. Previous studies prove that the AB algorithm is one of the appropriate options for many medical diagnosis applications (Saad, Khadour & Kanafani, 2016). The AB classification equation can be represented as

(2) $$f(g) = sign \bigg (\sum\limits_{m = 1}^M {\theta _m}{f_m}(g) \bigg)$$

where *f*_*m*_ is the *m*^*th*^ weak classifier and is the corresponding weight.

The hyper-parameter settings are shown in Table 4 for the Adaboost model, the decision tree as weak learners with max_depth 15 has been used, which means the tree can grow to a maximum of 15 level depth. Similarly, random_parameter is used every time as a seed to the random number generator incorporated in the algorithm. The value set for n_estimator is 500 which shows the number of weak learners for iterative training. The 0.8 learning rate has been used which indicates the weights of weak learners (Mathanker et al., 2011; Schapire, 2013).

**Table 4:** Model parameters setting.

Classifier	Hyper-parameters
AB	n estimators = 500, random state = 2,l earning rate = 0.8, base estimator = Decision Tree (max depth = 15)
RF	n estimators = 500, random state = 2, max depth = 15, criterion = ’en- tropy’
ETC	n estimators = 50, random state = 2, max depth = 15, criterion = ’en- tropy’
LR	random state = 2, solver = ‘liblinear, C = 2.0

#### Random forest

Random forest is considered one of the most popular and widely used ensemble algorithm. Several experiments carried out on medical datasets in Alam, Rahman & Rahman (2019) prove the significant supremacy of RF classifier over other classifiers like SVM, multilayer perceptron, and Bayes network, etc. This algorithm creates a forest with a large number of decision trees, the more the trees are in the forest, the more robust the prediction is. In RF, multiple decision trees are used to predict a class (Rustam et al., 2020). Each tree gives a prediction class based on the attributes which mean that the tree is giving a vote in support of a particular class. The forest finally chooses the class with the most votes over other predicted classes. RF thus produces relatively accurate predictions by joining many decision trees. The bagging technique is used in RF to train the decision trees using bootstrap sampling. A bootstrap sample is acquired from sub-samples of the training dataset with replacements, the size of each sample is however same as the training dataset. Thus the RF final prediction *p* is produced by a majority of votes given by decision trees in the forest and represented as

(3) $$prediction = mode\{ {T_1}(x),{T_2}(x),...,{T_2}(x)\} $$

(4) $$prediction = mode\{ \sum\limits_{n = 1}^N {T_n}(x)\}$$

where *T*_1_(*x*),*T*_2_(*x*),…,*T*_*n*_(*x*) are the number of decision trees in the RF model.

The current study uses RF with four hyperparameters as given in Table 3. The n_estimator has been used as 500 indicating the number of decision trees to give a prediction on a single record which are later used for the final prediction through majority voting. The max_depth value is 15 which indicates that each tree can grow to a 15 level depth. Another parameter criterion has been used with an ‘entropy’ setting which measures the impurity of a node in a decision tree (Dagum, 1997).

#### Extra trees classifier

Extra tree classifier also known as extremely randomized trees classifier is another ensemble modeling technique used in this study. ETC is very similar to the RF, wherein the model collects the outcomes of multiple de-correlated decision trees of a forest to produce its final classification outcome. The difference between the two is the way the decision trees’ are constructed in the forest from the original training sample. Each decision tree at every test node is given a random sample of *k* attributes from the attribute set. So the two main differences are splitting nodes by choosing fully random cut-points and usage of the learning sample as a whole (Geurts, Ernst & Wehenkel, 2006; Rustam et al., 2019). The selection of the best attribute for the split point is usually based on the Gini index or entropy value. Thus multiple numbers of de-correlated trees are created using a random sample of attributes. For ETC, the same parameter settings have been used as in the RF as shown in Table 3.

#### Logistic regression

The name LR has been derived from the statistical logistic function on which the method is based on. The logistic function is also known as the Sigmoid function in the LR model is used to map predictions to probabilities (Rustam et al., 2020). LR is a linear model and in our study and it is the only algorithm that is not an ensemble or tree-based classifier. LR classifier is more appropriate when the attribute data is numerical and the target is categorical as is the case with the current study. Recently the algorithm has been used in numerous medical research areas including etiology, epidemiology, and discrimination modeling in clinical diagnosis, etc (Yao et al., 2011). To map predicted values to probabilities, the Sigmoid function is used as

(5) $$f(g) = \displaystyle{L \over {1 + {e^{ - n(x - {x_o})}}}}$$

where 1 is the maximum value of the curve, *m* is the logistic growth rate, and *v*_0_ is the x-value of the sigmoid midpoint. Three parameters including solver, *C*, and random_state have been used in this study. Solvers are the algorithms that are used for the optimization and ‘liblinear’ has been used for the experiments in the current study because it performs better on a small dataset. The *C* is the inverse of regularization strength and its value is set to 2.0 which specifies the strength of regularization (Genkin, Lewis & Madigan, 2007).

### Proposed methodology

**Figure 5:**
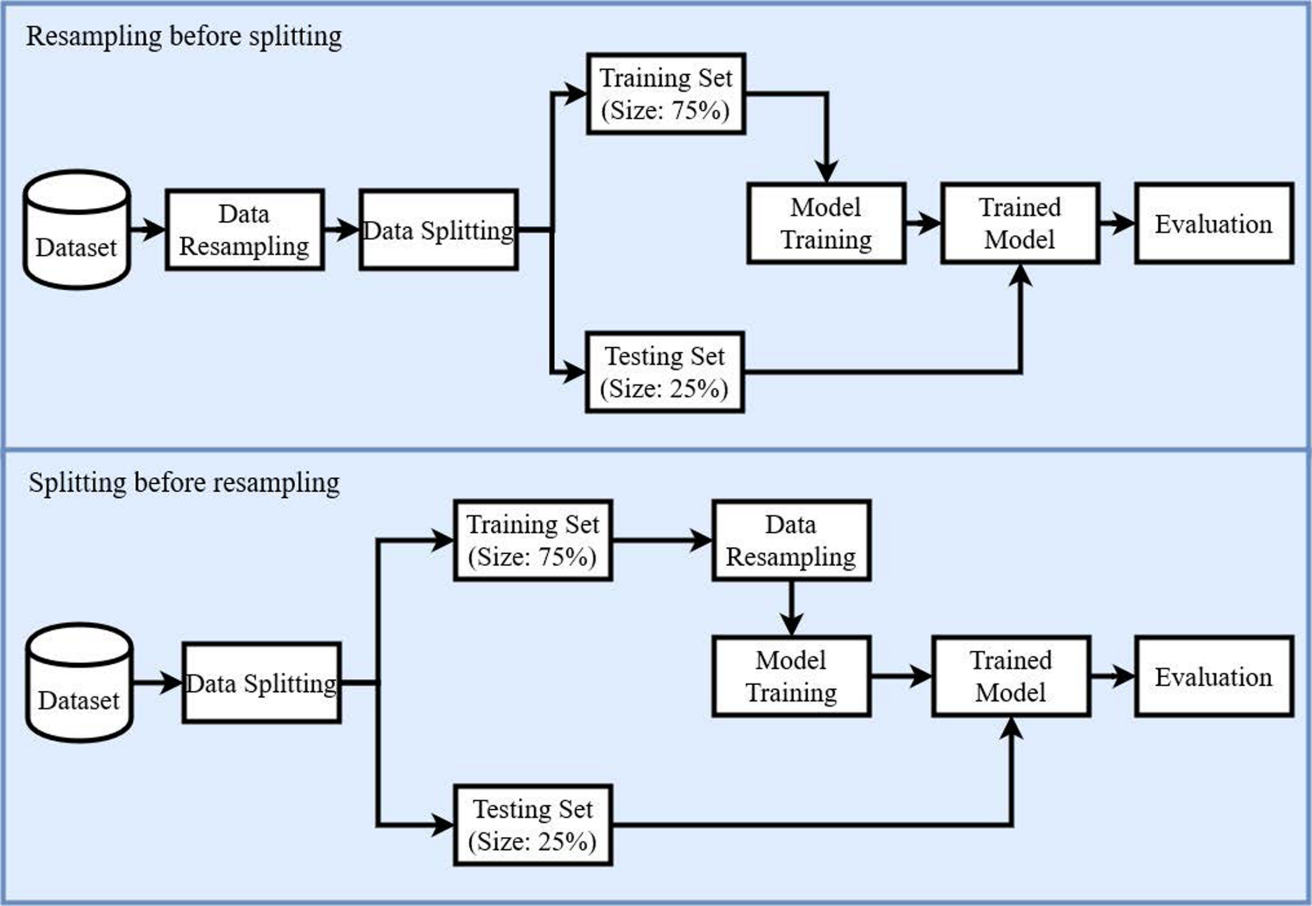
Diagram of the proposed methodology.

In this study different machine learning techniques have been used to solve the pathologies classification problem. Specifically, four supervised machine learning models have been used such as AB, RF, ETC, and LR with and without re-sampling techniques. The workflow of the model construction is shown in Fig. 5. In the first phase, the dataset has been re-sampled by using re-sampling techniques to balance the given dataset. The record count for each class after applying CC, SMOTE, ADASYN, and CR are shown in Table 5. All re-sampling techniques have been used with their default parameter settings that is the reason ADASYN generates 147 records for normal patients class.

**Table 5:** The record count for each class after applying CC, SMOTE, ADASYN and CR.

Records type	Before re-sampling	After sampling			
		CC	SMOTE	ADASYN	CR
Spondylolisthesis	150	60	150	150	360
Disc herniation	60	60	150	150	360
Normal	100	60	150	147	357
Total records	310	180	450	447	1,077

After balancing the dataset by re-sampling, all the records have been shuffled, and the dataset has been split into training and testing subsets. The split ratio of training and testing subsets has been set as 75:25 respectively. All experiments have been conducted using Core i7 7th generation machine on the Microsoft Windows 10 platform in the Python language using Anacondas 3 with a Jupyter notebook.

### Performance evaluation metrics

Several evaluation metrics are used to analyze the performance of the selected classifiers and data re-sampling methods. These metrics are discussed briefly in the following sections.

**Figure 6:**
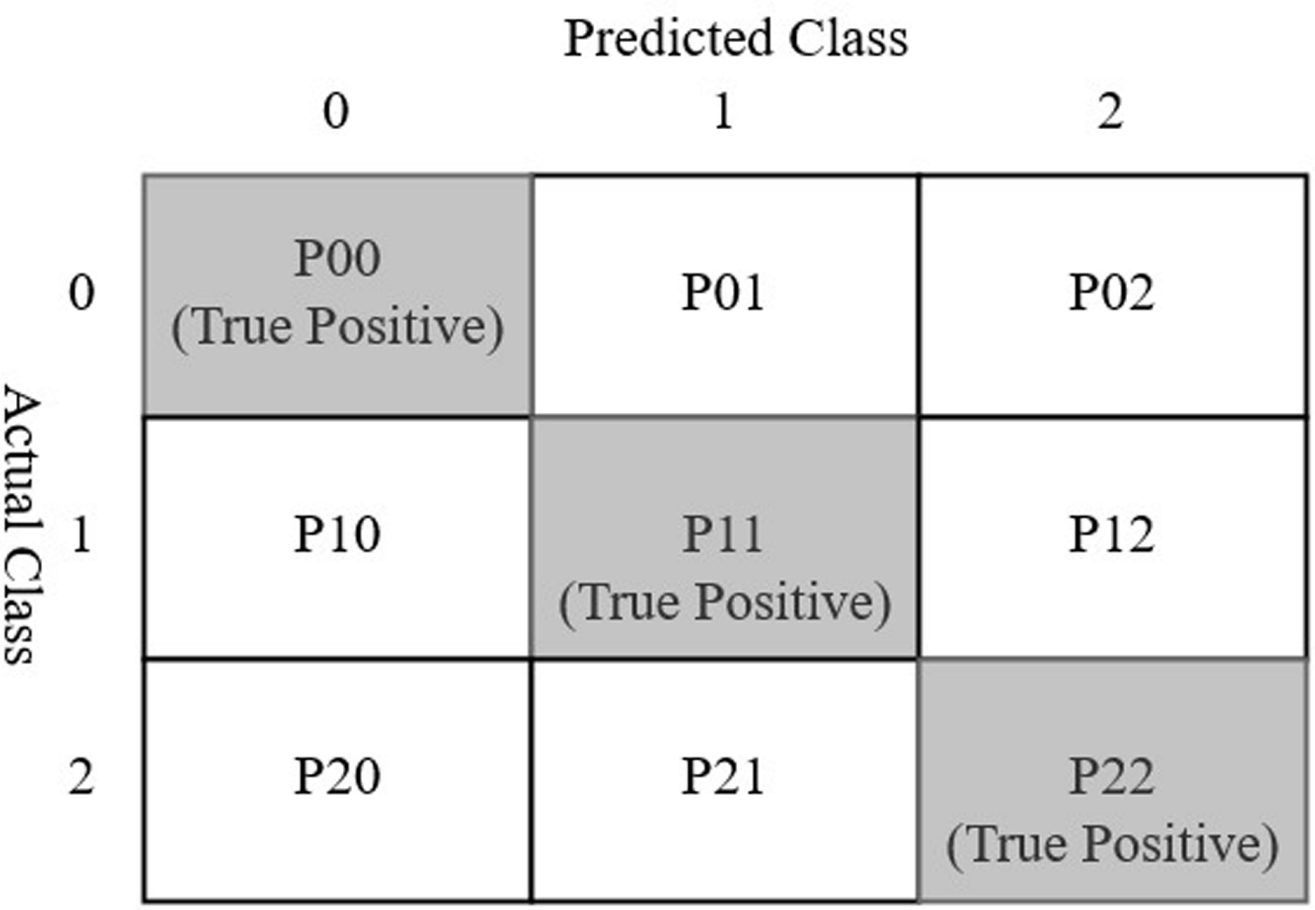
Confusion matrix for three class prediction results used in the current study.

A confusion matrix is a table layout used to visualize the performance of a classification model using a test data set for which the actual values are known (Wisdom, 2018; Data School, 2015; Rustam et al., 2020). The typical layout of a confusion matrix is given in Fig. 6. Considering three classes spondylolisthesis, normal, and hernia represented by class 0, 1, and 2 respectively, each cell of the confusion matrix can be described as follows

- **P00:** The cell value shows the number of predictions where class 0 was correctly classified (as class 0). The value represents the true positive (TP) for class 0.
- **P01:** The cell shows the number of predictions where class 1 was incorrectly classified as class 0.
- **P02:** This cell shows the number of predictions where class 2 was incorrectly classified as class 0.
- **P10:** The cell shows the number of predictions where class 0 was incorrectly classified class 1.
- **P11:** The cell shows the number of predictions where class 1 was correctly classified (as class 1). This cell represents TP for class 1.
- **P12:** The cell shows the number of predictions where class 2 was incorrectly classified as class 1.
- **P20:** The cell shows the number of predictions where class 0 was incorrectly classified as class 2.
- **P21:** The cell shows the number of predictions where class 1 was incorrectly classified as class 2.
- **P22:** The cell shows the number of predictions where class 2 were correctly classified (as class 2). The cell represents TP for class 2.

The accuracy of a model is calculated as the ratio of the number of model’s right predictions to the total number of predictions. So in line with the above confusion matrix notations, accuracy can be defined as:

(6) $$Accuracy = \displaystyle{{TP + TN} \over {TP + TN + FP + FN}}$$

where

- **TP (true positive):** The number of instances in which a model predicted a particular class out of the three classes (Spondylolisthesis or Hernia or normal) correctly.
- **TN (true negative):** The number of instances in which a model predicted a class as negative which is negative.
- **FP (false positive):** False positive or type 1 error is the number of cases where a model predicted a class as positive and it is negative. For Example, the number of predictions where persons having no herniation was incorrectly classified as hernia cases.
- **FN (false negative):** False-negative or Type 2 error is the number of cases where a model predicted a class as negative which is positive. For Example, the number of predictions where persons having herniation was incorrectly classified as another case Le et al. (2020b).

Following the four parameters defined above, accuracy, precision, recall and *F*_1_ score are defined using the following equations (Le & Nguyen, 2019)

(7) $$Precision = \displaystyle{{TP} \over {TP + FP}}$$

(8) $$Recall = \displaystyle{{TP} \over {TP + FN}}$$

(9) $${F_1}Score = 2 \times \displaystyle{{Precision \times Recall} \over {Precision + Recall}}$$

## Results and discussion

The current study considers several machine learning algorithms for the diagnosis of vertebral column pathology. Different re-sampling approaches have been shown to enhance the accuracy of the machine learning classifiers for predicting various diseases. To analyze the influence of re-sampling approaches for disc pathologies, this study focuses on the use of two well-known re-sampling approaches under391 sampling and over-sampling. By analyzing their influence on the ML algorithms, this study contrives a novel approach to further elevate their performance. Consequently, a novel resampling approach called concatenated resampling is proposed for improving the accuracy of ML algorithms when trained with an imbalanced dataset. Performance analysis is carried out to show the supremacy of the proposed CR approach.

The experiments have been conducted on the dataset obtained from the UCI data repository. Concerning the data imbalance, several data re-sampling approaches are considered as well to enhance the performance of the selected classifiers. So, experiments are carried for the following scenarios

- Performance of the classifiers on the dataset without re-sampling.
- Performance analysis using data under-sampling techniques.
- Classifiers’ performance on the balanced dataset with over-sampling techniques.
- Analysis of classifiers’ performance with the proposed concatenated re-sampling approach.

Initially, all models have been trained on the original dataset (imbalanced dataset) to check the performance of learning models. Later, experiments have been conducted by applying re-sampling techniques such as ADASYN, SMOTE, CC, and the novel CR to balance the dataset. The resulted dataset is then used for the training and testing of all models.

### Performance without dataset re-sampling

The selected classification algorithms are initially tested with the imbalanced dataset to analyze the performance. Table 6 shows the results of these models without using data re-sampling approaches. Results indicate that the performance of the RF classifier is superior to other classifiers under investigation. The performance of other classifiers is almost similar regarding accuracy. Similarly, the values for precision, recall, and *F*_1_ scores for these classifiers are very close indicating their similar performance with the imbalanced dataset.

**Table 6:** Performance results without re-sampling.

Classifier	Accuracy	Class	Precision	Recall	*F*_1_ Score
RF	0.88	Hernia	0.83	0.71	0.77
		Normal	0.87	0.87	0.87
		Spondylolisthesis	0.92	0.96	0.94
		Macro avg.	0.87	0.85	0.86
		Weighted Avg.	0.88	0.87	0.88
LR	0.83	Hernia	0.70	0.50	0.58
		Normal	0.81	0.83	0.82
		Spondylolisthesis	0.90	0.97	0.93
		Macro avg.	0.81	0.77	0.78
		Weighted Avg.	0.83	0.83	0.83
ETC	0.83	Hernia	0.82	0.64	0.72
		Normal	0.82	0.77	0.79
		Spondylolisthesis	0.86	0.97	0.90
		Macro avg.	0.83	0.79	0.81
		Weighted Avg.	0.84	0.83	0.83
AB	0.82	Hernia	0.75	0.64	0.69
		Normal	0.81	0.80	0.80
		Spondylolisthesis	0.87	0.91	0.89
		Macro avg.	0.80	0.78	0.79
		Weighted Avg.	0.83	0.82	0.82
SVM	0.85	Hernia	0.64	0.54	0.58
		Normal	0.77	0.80	0.78
		Spondylolisthesis	0.95	0.97	0.96
		Macro avg.	0.79	0.77	0.78
		Weighted Avg.	0.84	0.85	0.84

**Figure 7:**
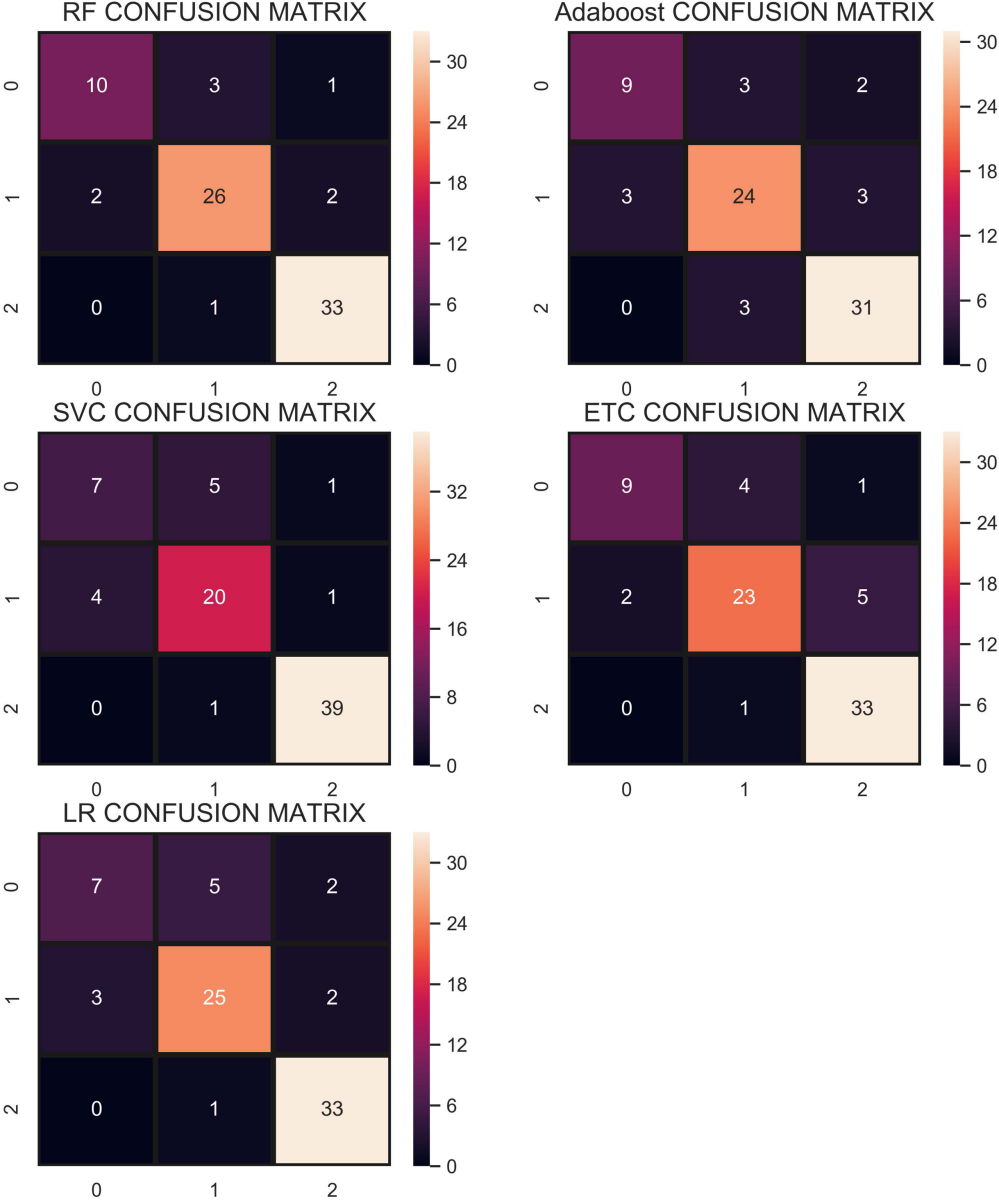
Confusion matrix of each classifier when trained without re-sampling.

The confusion matrix shown in Fig. 7 indicates that RF gives the highest number of correct predictions than that of other classifiers. RF gives 69 correct predictions out of 78 and only 9 predictions are wrong. The highest number of wrong predictions is given by the AB classifier which gives 14 wrong predictions and 64 correct predictions out of 78 predictions. So, RF is the outperformer while AB is the poor performer while LR, ETC show the same performance concerning the accuracy, recall, and *F*_1_ score but show little difference in precision.

### Performance using CC under-sampling technique—strategy 1

**Figure 8:**
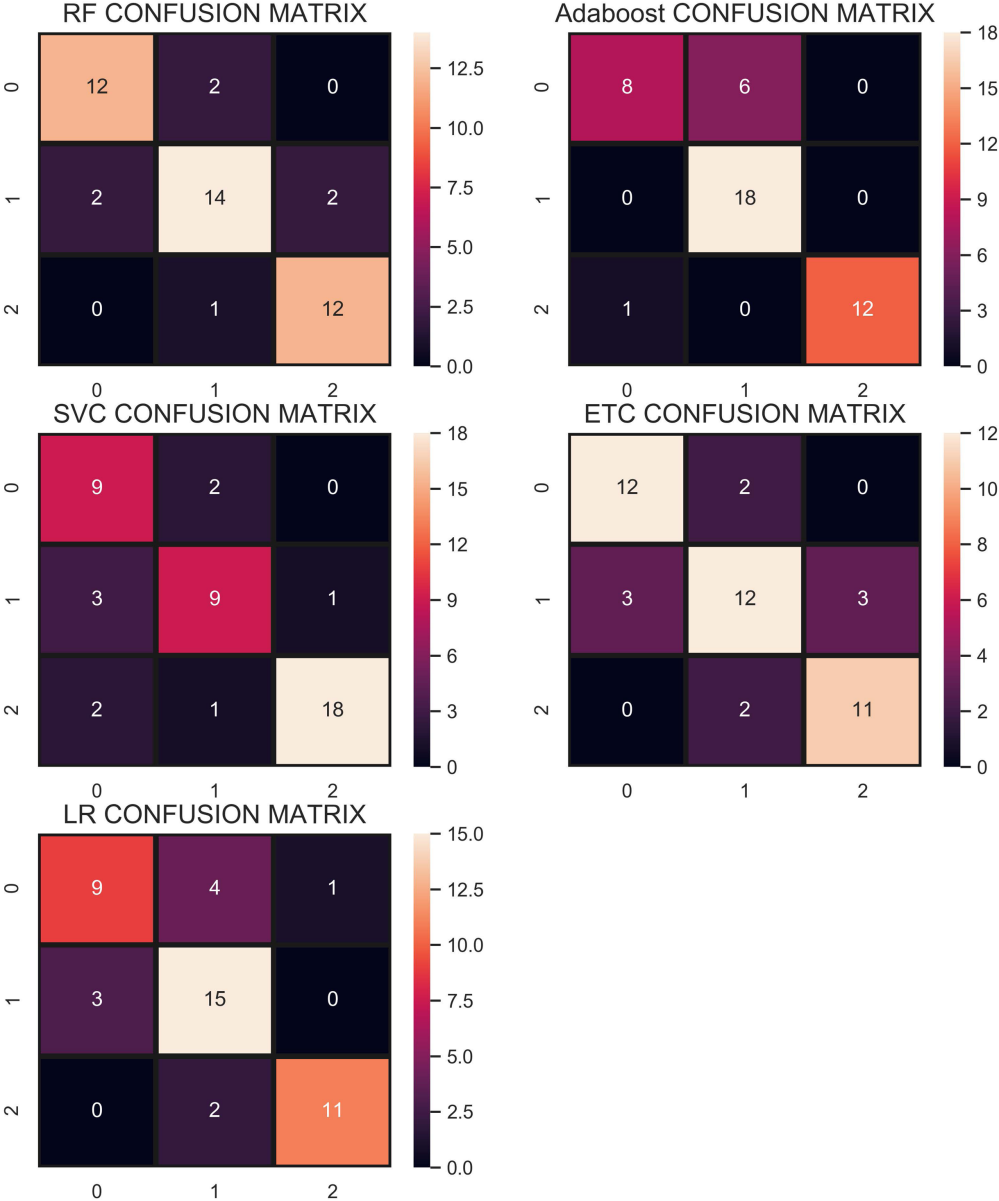
Confusion matrix for each classifier when trained and tesedt using CC under-sampling technique.

In the under-sampling technique, CC has been used to balance the dataset. CC under-sampling resulted in a total of 180 records which were split into 135 and 45 for training and testing, respectively. Table 7 and the confusion matrix in Fig. 8 show the performance results of all models when CC under-sampling is applied to balance the dataset. Results reveal that RF and AB classifiers give the highest accuracy of 0.84. The accuracy of ETC is 0.80, while LR performs poorly with the accuracy of 0.78 in this context. RF and Adaboost are the best performers while ETC is close to them in terms of performance while LR shows the lowest performance.

**Table 7:** Performance results with CC under-sampling technique.

Classifier	Accuracy	Class	Precision	Recall	*F*_1_ Score
RF	0.84	Hernia	0.86	0.86	0.86
		Normal	0.82	0.78	0.82
		Spondylolisthesis	0.86	0.92	0.89
		Macro avg.	0.85	0.85	0.85
		Weighted Avg.	0.84	0.84	0.84
LR	0.78	Hernia	0.75	0.64	0.69
		Normal	0.71	0.83	0.77
		Spondylolisthesis	0.92	0.85	0.88
		Macro avg.	0.79	0.77	0.78
		Weighted Avg.	0.78	0.78	0.78
ETC	0.80	Hernia	0.86	0.86	0.86
		Normal	0.76	0.72	0.74
		Spondylolisthesis	0.79	0.85	0.81
		Macro avg.	0.80	0.81	0.80
		Weighted Avg.	0.80	0.80	0.80
AB	0.84	Hernia	0.89	0.57	0.70
		Normal	0.75	1.00	0.86
		Spondylolisthesis	1.00	0.92	0.96
		Macro avg.	0.88	0.83	0.84
		Weighted Avg.	0.87	0.84	0.84
SVM	0.80	Hernia	0.64	0.82	0.72
		Normal	0.75	0.69	0.72
		Spondylolisthesis	0.95	0.86	0.90
		macro avg	0.78	0.79	0.78
		weighted avg	0.82	0.80	0.80

Overall, the performance of the classifiers has been reduced when trained on under-sampled datasets except for AB whose performance has been slightly improved. Primarily, the less number of records for each class resulted in degraded performance. According to Fig. 8, RF and AB give an equal number of correct predictions when trained and tested with an under-sampled dataset. RF and AB give 38 correct and 7 wrong predictions out of 45 predictions. LR gives 35 correct predictions which are less than all other models and 10 wrong predictions out of 45 predictions.

### Performance using ADASYN and SMOTE over-sampling techniques—strategy 1

#### SMOTE

Besides the under-sampling approach, two over-sampling approaches including ADASYN and SMOTE are also utilized to analyze their efficacy. SMOTE generated 450 records after over-sampling of which 337 and 113 were used for training and testing, respectively. The selected classifiers are trained and tested on the over-sampled dataset and results are given in Table 8. Results suggest that ETC performs the best among the selected classifiers when trained on the over-sampled dataset. For an over-sampled dataset, the accuracy of ETC can go up to 0.90 which is the highest among all classifiers. AB shows poor performance in this scenario as well with an accuracy of 0.76. The performance of classifiers is superior when an over-sampled dataset is used than that of an under-sampled dataset.

**Table 8:** Performance results with SMOTE.

Classifier	Accuracy	Class	Precision	Recall	*F*_1_ Score
RF	0.86	Hernia	0.79	0.91	0.85
		Normal	0.91	0.71	0.80
		Spondylolisthesis	0.93	1.00	0.96
		Macro avg.	0.88	0.88	0.87
		Weighted Avg.	0.87	0.86	0.86
LR	0.79	Hernia	0.78	0.70	0.74
		Normal	0.71	0.76	0.74
		Spondylolisthesis	0.93	1.00	0.96
		Macro avg.	0.81	0.82	0.81
		Weighted Avg.	0.79	0.79	0.79
ETC	0.90	Hernia	0.83	0.98	0.90
		Normal	0.97	0.76	0.85
		Spondylolisthesis	0.96	1.00	0.98
		Macro avg.	0.92	0.91	0.91
		Weighted Avg.	0.91	0.90	0.90
AB	0.76	Hernia	0.68	0.85	0.76
		Normal	0.77	0.55	0.64
		Spondylolisthesis	0.92	0.96	0.94
		Macro avg.	0.79	0.79	0.78
		Weighted Avg.	0.77	0.76	0.75
SVM	0.89	Hernia	0.85	0.86	0.86
		Normal	0.88	0.85	0.86
		Spondylolisthesis	0.97	0.97	0.97
		Macro avg.	0.90	0.90	0.90
		Weighted Avg.	0.89	0.89	0.89

**Figure 9:**
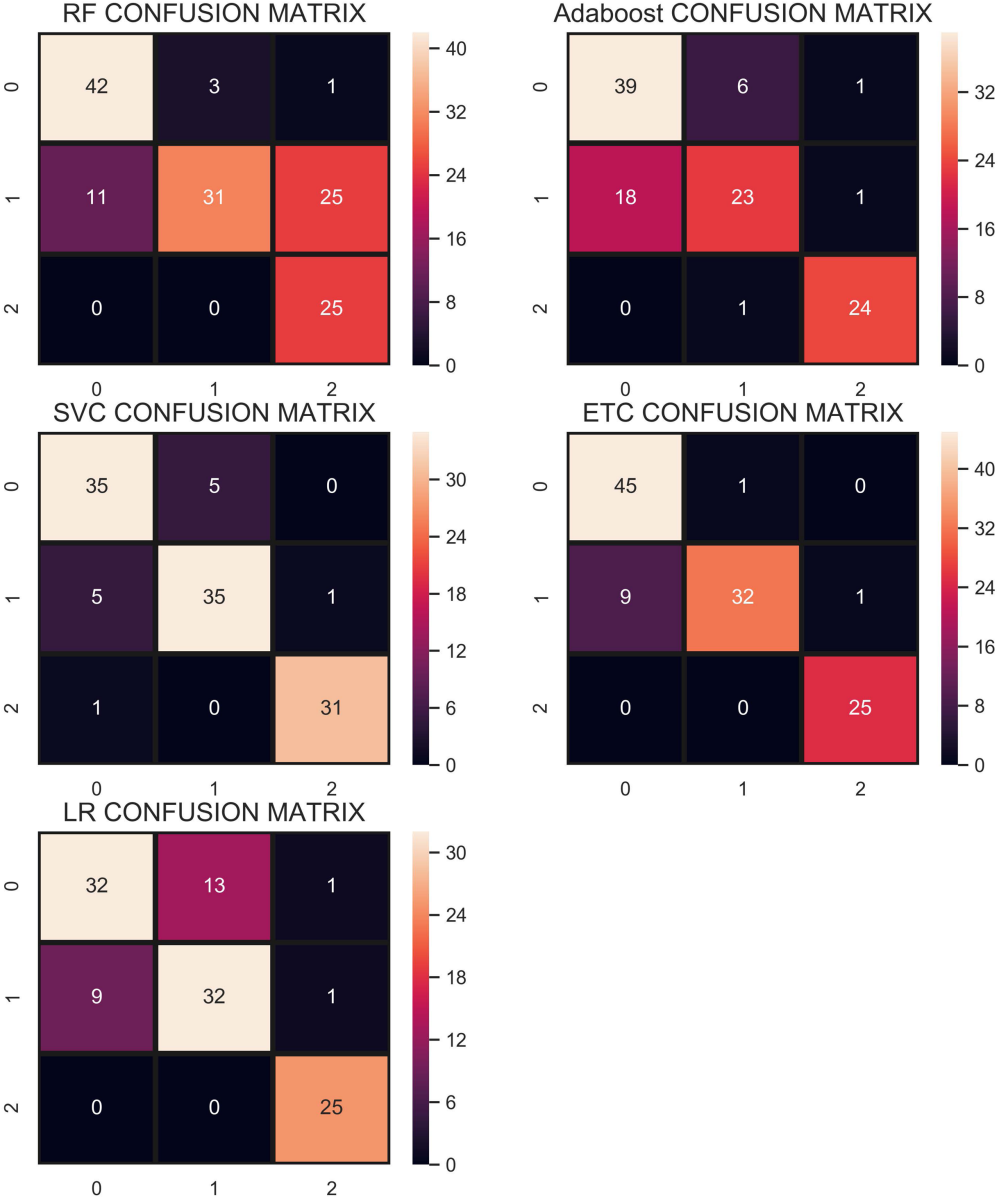
Confusion matrix showing classification results using the SMOTE technique.

The confusion matrix in Fig. 9 shows that the performance of ETC is superior when the SMOTE technique is used than that of CC and without re-sampling. In the SMOTE case, ETC correctly classifies 104 out of 113 test samples and only 9 predictions are wrong.

#### ADASYN

Another over-sampling technique ADASYN has been used in this study as well. ADASYN generates 447 instances after the over-sampling of the dataset. Dataset then has been divided into training and testing sets with the same ratio of 75:25 as we did it for SMOTE re-sampling techniques. Consequently, 335 records are used for training the models while 112 are used for model testing. Experimental results are shown in Table 9 which indicate that ADASYN re-sampling technique helps to improve classification accuracy. ETC achieves the highest accuracy of 0.95. Other than that, the performance of RF and AB has been elevated concerning the accuracy and other metrics. The average performance of all the classifiers is higher with the ADASYN over-sampling technique than that of CC under-sampling and SMOTE over-sampling. However, there is no change in the performance of LR.

**Table 9:** Performance results using the ADASYN over-sampling technique.

Classifier	Accuracy	Class	Precision	Recall	*F*_1_ Score
RF	0.93	Hernia	0.91	0.93	0.92
		Normal	0.95	0.88	0.91
		Spondylolisthesis	0.93	1.00	0.96
		Macro avg.	0.93	0.94	0.93
		Weighted Avg.	0.93	0.93	0.93
LR	0.79	Hernia	0.85	0.63	0.72
		Normal	0.69	0.85	0.76
		Spondylolisthesis	0.93	1.00	0.96
		Macro avg.	0.82	0.83	0.82
		Weighted Avg.	0.81	0.79	0.79
ETC	0.95	Hernia	0.96	0.96	0.96
		Normal	0.93	0.95	0.94
		Spondylolisthesis	0.96	0.92	0.94
		Macro avg.	0.95	0.94	0.95
		Weighted Avg.	0.95	0.95	0.95
AB	0.87	Hernia	0.84	0.91	0.87
		Normal	0.86	0.78	0.82
		Spondylolisthesis	0.92	0.92	0.92
		Macro avg.	0.87	0.87	0.87
		Weighted Avg.	0.87	0.87	0.87
SVM	0.79	Hernia	0.68	0.79	0.73
		Normal	0.76	0.63	0.69
		Spondylolisthesis	0.94	0.97	0.96
		Macro avg.	0.80	0.80	0.79
		Weighted Avg.	0.79	0.79	0.78

**Figure 10:**
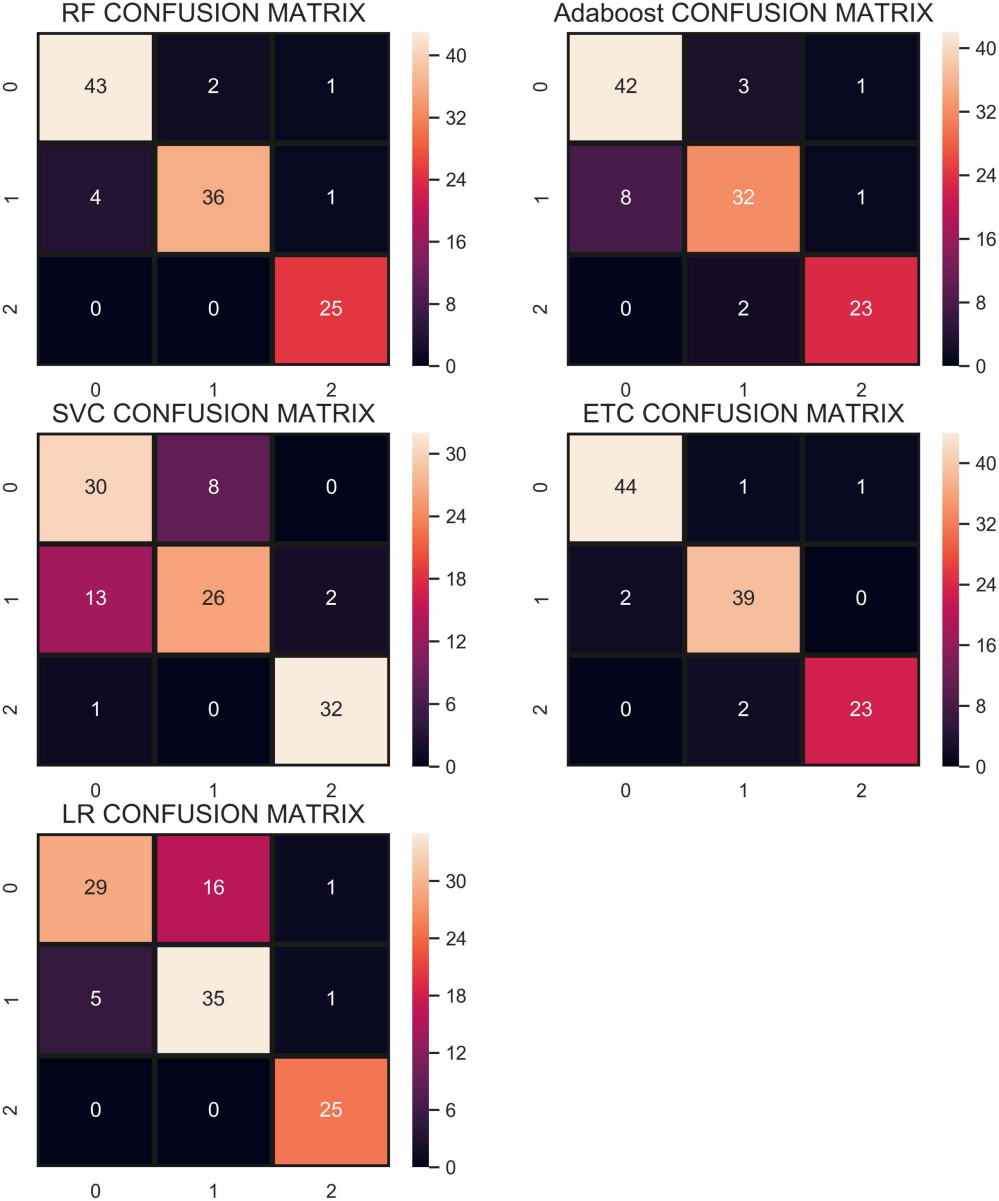
Confusion matrix for each classifier when trained using over-sampled data with the ADASYN technique.

Figure 10 shows the confusion matrix for selected classifiers. ETC performs very well and correctly classifies 106 samples out of 112. RF also performs well with a 0.93 accuracy score and gives 104 correct predictions. The classifier with the least performance in terms of accuracy and other metrics is LR with an accuracy of 0.79.

### Performance using concatenate re-sampling technique—strategy 1

We propose the use of a concatenated re-sampling approach which is a combination of ADASYN, SMOTE, and CC. For the CR approach, the results of ADASYN, SMOTE, and CC techniques have been concatenated which resulted in the enlarged dataset of 1,077 records. Thus, after splitting the dataset into training and testing sets, 807 records are used for the training while 270 are used for testing. Experimental results are shown in Table 10 which indicates the performance of all the classifiers is enhanced. ETC achieves the highest accuracy score of 0.99 and other tree-based models also perform very well. AB achieves its highest accuracy of 0.95 for the current problem and RF also achieves a 0.98 accuracy score. Accuracy of LR is elevated as well from 0.79 in ADASYN re-sampling to 0.85 in CR re-sampling.

**Table 10:** Performance results with CR.

Classifier	Accuracy	Class	Precision	Recall	*F*_1_ Score
RF	0.98	Hernia	0.96	0.99	0.98
		Normal	0.98	0.96	0.97
		Spondylolisthesis	1.00	0.99	0.99
		Macro avg.	0.98	0.98	0.98
		Weighted Avg.	0.98	0.98	0.98
LR	0.85	Hernia	0.86	0.82	0.84
		Normal	0.79	0.84	0.81
		Spondylolisthesis	0.96	0.95	0.95
		Macro avg.	0.87	0.87	0.87
		Weighted Avg.	0.86	0.86	0.86
ETC	0.99	Hernia	0.98	0.99	0.99
		Normal	0.99	0.99	0.99
		Spondylolisthesis	1.00	0.99	0.99
		Macro avg.	0.99	0.99	0.99
		Weighted Avg.	0.99	0.99	0.99
AB	0.95	Hernia	0.89	0.98	0.93
		Normal	0.98	0.87	0.92
		Spondylolisthesis	1.00	0.99	0.99
		Macro avg.	0.95	0.95	0.95
		Weighted Avg.	0.95	0.94	0.94
SVM	0.80	Hernia	0.64	0.82	0.72
		Normal	0.75	0.69	0.72
		Spondylolisthesis	0.95	0.86	0.90
		Macro avg.	0.78	0.79	0.78
		Weighted Avg.	0.82	0.80	0.80

**Figure 11:**
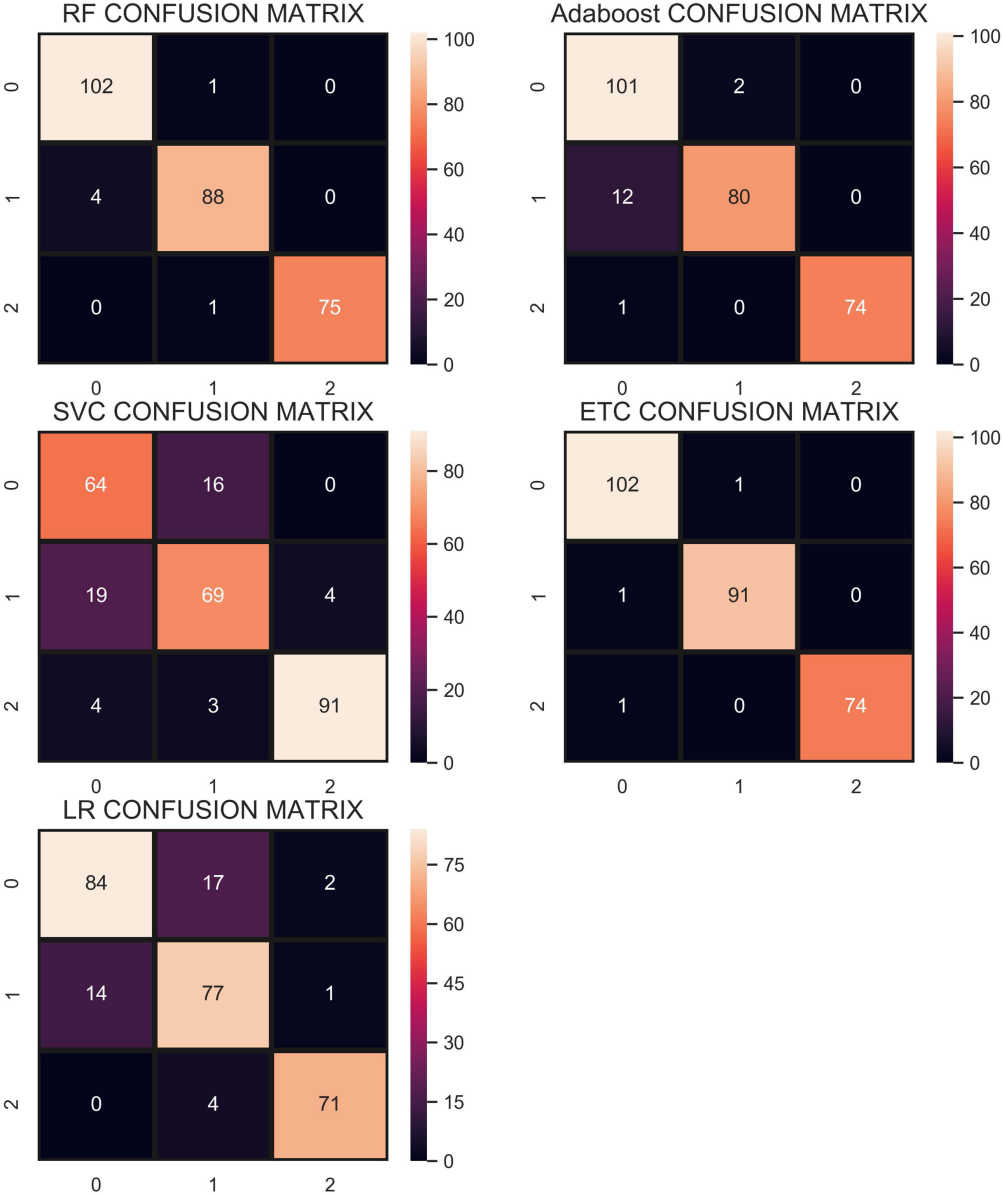
Confusion matrix for each classifier when trained using the CR technique.

The confusion matrix in Fig. 11 shows the performance of learning models with the CR re-sampling technique. ETC gives the highest number of correct predictions as compared to other models. Out of 270 test samples, ETC provides 267 correct predictions and only 3 wrong predictions. The proportion of the number of correct predictions shows how effective CR re-sampling is on small and imbalanced datasets. ETC and RF are the best performers while Adaboost is close to them in terms of performance. Results show that collectively the tree-based models are more effective in the given scenario.

### Performance using re-sampling technique—strategy 2

For strategy 2, the data re-sampling is carried once the data are split into training and testing data. Table 11 shows the accuracy, precision, recall, and F1 score of all the classifiers using CC undersampling. Results show change in the accuracy of different classifiers, e.g., RF from 0.84 to 0.81, LR 0.78 to 0.84, ETC 0.80 to 0.81, Adaboost 0.84 to 0.79, and SVM 0.82 to 0.90 using strategy 2. Consequently, the performance of LR and SVM has been elevated substantially.

**Table 11:** Performance results using CC sampling.

Classifier	Accuracy	Precision	Recall	*F*_1_ Score
RF	0.81	0.81	0.81	0.81
LR	0.84	0.84	0.84	0.84
ETC	0.81	0.81	0.81	0.81
AB	0.79	0.84	0.79	0.79
SVM	0.90	0.94	0.90	0.91

**Table 12:** Performance results using SMOTE sampling.

Classifier	Accuracy	Precision	Recall	*F*_1_ Score
RF	0.81	0.81	0.81	0.81
LR	0.86	0.81	0.82	0.81
ETC	0.85	0.84	0.85	0.84
AB	0.86	0.86	0.86	0.85
SVM	0.87	0.87	0.87	0.87

The performance of the classifiers using SMOTE is shown in Table 12. Results indicate that the performance of the classifiers has been changed slightly. For example, the accuracy of RF, ETC has been reduced to 0.81, and 0.85 from 0.86, and 0.90, respectively. However, the performance of LR and Adaboost is boosted when following strategy 2.

**Table 13:** Results using ADASYN sampling for strategy 2.

Classifier	Accuracy	Precision	Recall	*F*_1_ Score
RF	0.85	0.84	0.85	0.84
LR	0.87	0.88	0.87	0.88
ETC	0.83	0.83	0.83	0.83
AB	0.77	0.77	0.77	0.77
SVM	0.90	0.91	0.90	0.90

**Table 14:** Performance of CR resampling with strategy 2.

Classifier	Accuracy	Precision	Recall	*F*_1_ Score
RF	0.86	0.86	0.86	0.86
LR	0.93	0.93	0.92	0.92
ETC	0.83	0.83	0.83	0.83
AB	0.79	0.79	0.79	0.79
SVM	0.89	0.89	0.89	0.89

Similarly, Table 13 shows the results for performance evaluation parameters when predictions are made using ADASYN oversampling approach. Results suggest that the performance of RF, ETC, and Adaboost has been degraded substantially when strategy 2 is adopted. On the other hand, the performance of LR and SVM has been improved following this strategy. Using sampling before data split may result in having duplicate samples in both the training and test datasets which could overstate the performance of the classifiers. So when the data split is performed before the resampling, the performance is reduced slightly.

In the end, the experiments are performed using the proposed CR resampling approach to analyze if the results are affected when strategy 2 is adopted. Results shown in Table 14 prove that the accuracy of all the classifiers has been changed. For example, the accuracy of RF has been degraded substantially from 0.98 to 0.86 when strategy 2 is adopted. Similarly, the performance of ETC and Adaboost is reduced as well. Conversely, the performance of LR is greatly improved and the same is true for SVM. Despite the change in the accuracy of different classifiers, the overall performance of the classifiers is better when used with the proposed CR resampling approach.

### Performance comparison with other approaches

The efficacy of the proposed approach is analyzed by comparing its performance to several other similar approaches. For example, the study (Unal, Polat & Kocer, 2014) uses a combination of pairwise fuzzy C-means based feature weighting and SVM to classify samples into three classes of hernia, normal, and spondylolisthesis and achieves 96.4% accuracy. Similarly, the authors classified samples into normal and abnormal classes for vertebral column disorder in Unal, Polat & Kocer (2016) and achieved an accuracy of 99.3%. The classification has been done using the combination of MSCBAW (mean shift clustering-based attribute weighting) and RBDNN (radial basis function–neural network) in the study. To compare the performance of the proposed approach with Unal, Polat & Kocer (2016), ETC is trained on two classes with concatenated re-sampling. Hernia and spondylolisthesis class samples are merged into one class called, ‘abnormal’ while samples of healthy people belong to the ‘normal’ class. ETC achieves an accuracy of 99.5% when tested with two classes.

Performance results are given in Table 15. Results indicate that the proposed approach which comprises of ETC with CR re-sampling approach outperforms the state-of-the-art approaches for vertebral pathologies. The proposed approach can successfully classify the given samples into three classes of hernia, normal, and spondylolisthesis with high accuracy. So, the approach possesses the potential to assist medical experts in the accurate diagnosis of vertebral pathologies.

**Table 15:** Performance comparison with state-of-the-art approaches.

References	Target classes	Approach	Accuracy (%)
da Rocha Neto et al. (2011)	3	SVM	85.0
Karabulut & Ibrikci (2014)		LMT	89.0
Akben (2016)		MLP	83.0
Unal & Kocer (2013)		MLP	85.0
Alafeef et al. (2019)		ANN	98.5
Prasetio & Riana (2015)		KNN	93.0
Jiménez & Quintero-Ospina (2019)		Ensemble	90.0
Unal, Polat & Kocer (2014)		Fuzzy+SVM	96.4
Proposed		ETC with CR	**99.0**
Unal, Polat & Kocer (2016)	2	MSCBAW+RBDNN	99.3
Proposed		ETC with CR	**99.5**

**Note:**

Bold entries show the highest accuracy and indicate that the proposed approach outperforms state-of-the-art approaches.

To further corroborate the findings of this study performs a statistical T-test on the results of machine learning models and shows the significances of our proposed CR technique. We perform a T-test for both scenarios as follows:

- Resampling before splitting
- Splitting before resampling

We compare the model’s results to check that which technique is statistically significant for data resampling. To evaluate the T-test we take two hypotheses as:

1. Null hypothesis (NHo): The model results are statistically significant after apply CR as compare to SMOTE, ADASYN, and CC.
2. Alternative hypothesis (AHa): The model results are not statistically significant after apply CR as compare to SMOTE, ADASYN, and CC.

In the first scenario, tree-based models such as ETC, RF accept the NHo and reject AHa which means their performance is statistically significant after applying resampling techniques. On the other hand, for the second scenario, LR performs statically significant after applying CR as compared to other re-sampling techniques because it accepts the NHo hypothesis. These statistical T-test results show that the CR technique is better as compared to other individual re-sampling techniques.

Due to the lack of a similar dataset, cross-validation is used to confirm the performance of the proposed approach. Table 16 shows the results of 10-fold cross-validation which indicates the performance of the proposed resampling approach with all the selected classifiers.

**Table 16:** Results of machine learning classifiers using cross validation.

Model	CR Resampling		CC Resampling	
	Accuracy	Confidence Interval	Accuracy	Confidence Interval
RF	0.986	+/−0.013	0.794	+/−0.061
LR	0.850	+/−0.017	0.794	+/−0.093
ETC	0.990	+/−0.013	0.767	+/−0.082
AB	0.978	+/−0.017	0.756	+/−0.103
SVM	0.836	+/−0.038	0.828	+/−0.058
	SMOTE Resampling		ADASYN Resampling	
RF	0.911	+/−0.038	0.917	+/−0.046
LR	0.847	+/−0.066	0.803	+/−0.052
ETC	0.920	+/−0.041	0.909	+/−0.062
AB	0.873	+/−0.043	0.866	+/−0.056
SVM	0.840	+/−0.049	0.810	+/−0.071

## Conclusion

Machine learning classification techniques assist medical practitioners in several medical domains and pathology diagnosis is no exception. The classification of biomedical data is considered a very sensitive and challenging task. Considering the complexity and demand for continuous improvements in the automatic bio-medical classification, this paper is an effort to investigate three popular tree-based ensemble machine learning classifiers including AB, RF, ETC along with a famous probability-based non-tree classifier LR. The experiments have been carried out on a bio-medical dataset related to vertebral column pathology diagnosis. Initially, the selected algorithms are trained and tested on the imbalanced dataset to test the performance results of each model. Then re-sampling techniques including, under-sampling and over-sampling are utilized to balance the dataset. Thus, the study investigates several models with and without re-sampling techniques to compare the performance of various models and re-sampling techniques.

Experimental results indicate that the RF classifier performs better in two scenarios when models are trained without re-sampling, as well as when under-sampling is used in the training dataset. RF classifier is more robust to over-fitting when applied on im-balanced datasets and its parameterization remains quite intuitive. On the other hand, when over-sampling was applied to the same training dataset ETC showed better performance with an accuracy of 0.90 using SMOTE and 0.95 using ADASYN. It was also observed that the application of over-sampling on the given dataset improves the performance of each classifier compared to the under-sampling technique except for the AB classifier. Results also show that LR does not perform well on the datasets used in this study in all the scenarios considered in the current study.

Besides the experiment with under and over-sampling approaches, a novel re-sampling approach called concatenated re-sampling is proposed which integrates the output of CC under-sampling and SMOTE and ADASYN over-sampling approaches to make a balanced dataset which is later used for training and testing the performance of the selected classifiers. Results reveal that the classification performance of all the classifiers is elevated when trained on the dataset balanced by the proposed CR re-sampling approach. ETC achieves the highest accuracy of 0.99 when the CR technique was applied to the dataset. CR outperforms other re-sampling approaches because it increases the size of the data to the level where the models can learn better than individual re-sampling techniques. Results reveal that the combination of over-sampling and under-sampling can boost the performance of classifiers. In the future, we intend to consider recently developed models and methodologies for other datasets in diverse medical diagnostic applications.

## Supplemental Information

10.7717/peerj-cs.547/supp-1 Supplemental Information 1 Code associated with the paper.

## Additional Information and Declarations

### Data Availability

The following information was supplied regarding data availability: All the codes related to the implementation of machine learning models used in the current study are available in the Supplemental File.

## References

[ref-5] Akben SB (2016). Importance of the shape and orientation of the spine and pelvis for the vertebral column pathologies diagnosis with using machine learning methods. Biomedical Research-India (Special Issue on Health Science and Bio Convergence Technology).

[ref-6] Alafeef M, Fraiwan M, Alkhalaf H, Audat Z (2019). Shannon entropy and fuzzy c-means weighting for ai-based diagnosis of vertebral column diseases. Journal of Ambient Intelligence and Humanized Computing.

[ref-7] Alam MZ, Rahman MS, Rahman MS (2019). A random forest based predictor for medical data classification using feature ranking. Informatics in Medicine Unlocked.

[ref-8] Asai Y, Tsutsui S, Oka H, Yoshimura N, Hashizume H, Yamada H, Akune T, Muraki S, Matsudaira K, Kawaguchi H, Nakamura K, Tanaka S, Yoshida M, Smith J (2017). Sagittal spino-pelvic alignment in adults: the wakayama spine study. PLOS ONE.

[ref-9] Ayat N-E, Cheriet M, Remaki L, Suen CY (2001). Kmod-a new support vector machine kernel with moderate decreasing for pattern recognition. application to digit image recognition.

[ref-10] Baldit A (2018). Micromechanics of the intervertebral disk.

[ref-11] Chawla NV, Bowyer KW, Hall LO, Kegelmeyer WP (2002). Smote: synthetic minority over-sampling technique. Journal of Artificial Intelligence Research.

[ref-12] Chen P-HC, Liu Y, Peng L (2019). How to develop machine learning models for healthcare. Nature Materials.

[ref-13] da Rocha Neto AR, Sousa R, Barreto GDA, Cardoso JS (2011). Diagnostic of pathology on the vertebral column with embedded reject option.

[ref-2] Dataman in AI (2018). Using under-sampling techniques for extremely imbalanced data. Medium.

[ref-53] Data School (2015). Simple guide to confusion matrix terminology. https://www.dataschool.io/simple-guide-to-confusion-matrix-terminology.

[ref-14] Dagum C (1997). Decomposition and interpretation of gini and the generalized entropy inequality measures. STATISTICA-BOLOGNA.

[ref-4] DeSai C, Vamsi R, Agarwal A (2018). Anatomy back vertebral column. https://www.ncbi.nlm.nih.gov/books/NBK525969/.

[ref-16] Dennis B, Muthukrishnan S (2014). Agfs: adaptive genetic fuzzy system for medical data classification. Applied Soft Computing.

[ref-17] Duarte FR, Sousa AMSND, Raposo FJA, Valente LFA, Neves NSM, Gonçalves AM, Pinto RAP (2013). Impact of spino-pelvic balance on clinical and functional results after instrumented fusion in patients with degenerative spondylolisthesis. Coluna/Columna.

[ref-18] Fardon DF, Milette PC (2001). Nomenclature and classification of lumbar disc pathology: recommendations of the combined task forces of the north american spine society, american society of spine radiology, and american society of neuroradiology. Spine.

[ref-19] Genkin A, Lewis DD, Madigan D (2007). Large-scale bayesian logistic regression for text categorization. Technometrics.

[ref-20] Geurts P, Ernst D, Wehenkel L (2006). Extremely randomized trees. Machine learning.

[ref-21] Gorunescu F, Belciug S (2014). Evolutionary strategy to develop learning-based decision systems. application to breast cancer and liver fibrosis stadialization. Journal of Biomedical Informatics.

[ref-22] He H, Bai Y, Garcia EA, Li S (2008). Adasyn: adaptive synthetic sampling approach for imbalanced learning.

[ref-23] Ho Thanh Lam L, Le NH, Van Tuan L, Tran Ban H, Nguyen Khanh Hung T, Nguyen NTK, Huu Dang L, Le NQK (2020). Machine learning model for identifying antioxidant proteins using features calculated from primary sequences. Biology.

[ref-24] Huang M-W, Chen C-W, Lin W-C, Ke S-W, Tsai C-F (2017). Svm and svm ensembles in breast cancer prediction. PLOS ONE.

[ref-15] Jabbar MA, Deekshatulu BL, Chandra P (2013). Classification of heart disease using k-nearest neighbor and genetic algorithm. Procedia Technology.

[ref-25] Jiménez DR, Quintero-Ospina JD (2019). Classification of pathologies present in the spinal column through learning machinery techniques. Ingeniera Solidaria.

[ref-26] Karabulut EM, Ibrikci T (2014). Effective automated prediction of vertebral column pathologies based on logistic model tree with smote preprocessing. Journal of Medical Systems.

[ref-27] Le NQK, Do DT, Chiu F-Y, Yapp EKY, Yeh H-Y, Chen C-Y (2020a). Xgboost improves classification of mgmt promoter methylation status in idh1 wildtype glioblastoma. Journal of Personalized Medicine.

[ref-28] Le NQK, Ho Q-T, Yapp EKY, Ou Y-Y, Yeh H-Y (2020b). Deepetc: a deep convolutional neural network architecture for investigating and classifying electron transport chain’s complexes. Neurocomputing.

[ref-29] Le NQK, Nguyen V-N (2019). Snare-cnn: a 2d convolutional neural network architecture to identify snare proteins from high-throughput sequencing data. PeerJ Computer Science.

[ref-30] Léo JCD, Léo Álvaro CD, Cardoso IM, Jacob Júnior C, Batista Júnior Jé L (2015). Association of spinopelvic parameters with the location of lumbar disc herniation. Coluna/Columna.

[ref-31] Magee DJ, Zachazewski JE, Quillen WS, Manske RC (2015). Pathology and intervention in musculoskeletal rehabilitation.

[ref-32] Mandal I (2015). Developing new machine learning ensembles for quality spine diagnosis. Knowledge-Based Systems.

[ref-3] Marks H (2019). Types of spine curvature disorders. WebMD.

[ref-33] Mathanker S, Weckler P, Bowser T, Wang N, Maness N (2011). Adaboost classifiers for pecan defect classification. Computers and Electronics in Agriculture.

[ref-34] Prasetio RT, Riana D (2015). A comparison of classification methods in vertebral column disorder with the application of genetic algorithm and bagging.

[ref-35] Raciborski F, Gasik R, Kłak A (2016). Disorders of the spine: a major health and social problem. Reumatologia.

[ref-36] Rajpurkar P, Irvin J, Zhu K, Yang B, Mehta H, Duan T, Ding D, Bagul A, Langlotz C, Shpanskaya K, Lungren MP, Ng AY (2017). Chexnet: radiologist-level pneumonia detection on chest x-rays with deep learning. https://arxiv.org/abs/1711.05225.

[ref-37] Rustam F, Ashraf I, Mehmood A, Ullah S, Choi GS (2019). Tweets classification on the base of sentiments for us airline companies. Entropy.

[ref-38] Rustam F, Mehmood A, Ahmad M, Ullah S, Khan DM, Choi GS (2020). Classification of shopify app user reviews using novel multi text features. IEEE Access.

[ref-39] Saad G, Khadour A, Kanafani Q (2016). Ann and adaboost application for automatic detection of microcalcifications in breast cancer. The Egyptian Journal of Radiology and Nuclear Medicine.

[ref-40] Samb ML, Camara F, Ndiaye S, Slimani Y, Esseghir MA (2012). A novel rfe-svm-based feature selection approach for classification. International Journal of Advanced Science and Technology.

[ref-41] Schapire RE (2013). Explaining adaboost.

[ref-42] Seera M, Lim CP (2014). A hybrid intelligent system for medical data classification. Expert Systems with Applications.

[ref-43] Tenny S, Gillis CC (2019). Spondylolisthesis.

[ref-1] UCI Machine Learning Repository (2011). Vertebral column data set. http://archive.ics.uci.edu/ml/datasets/vertebral+column.

[ref-44] Unal Y, Kocer HE (2013). Diagnosis of pathology on the vertebral column with backpropagation and nave bayes classifier.

[ref-45] Unal Y, Polat K, Kocer HE (2014). Pairwise fcm based feature weighting for improved classification of vertebral column disorders. Computers in Biology and Medicine.

[ref-46] Unal Y, Polat K, Kocer HE (2016). Classification of vertebral column disorders and lumbar discs disease using attribute weighting algorithm with mean shift clustering. Measurement.

[ref-47] Vialle LR, Vialle EN, Henao JES, Giraldo G (2010). Lumbar disc herniation. Revista Brasileira de Ortopedia (English Edition).

[ref-48] Wiens J, Saria S, Sendak M, Ghassemi M, Liu VX, Doshi-Velez F, Jung K, Heller K, Kale D, Saeed M, Ossorio PN, Thadaney-Israni S, Goldenberg A (2019). Do no harm: a roadmap for responsible machine learning for health care. Nature Medicine.

[ref-49] Wisdom BD (2018). Understanding the confusion matrix (ii). https://dev.to/overrideveloper/understanding-the-confusion-matrix-264i.

[ref-50] Wroge TJ, Özkanca Y, Demiroglu C, Si D, Atkins DC, Ghomi RH (2018). Parkinson’s disease diagnosis using machine learning and voice.

[ref-51] Yao K-W, He Q-Y, Teng F, Wang J (2011). Logistic regression analysis of syndrome essential factors in patients with unstable angina pectoris. Journal of Traditional Chinese Medicine.

[ref-52] Yen S-J, Lee Y-S (2009). Cluster-based under-sampling approaches for imbalanced data distributions. Expert Systems with Applications.

